# Retraining and Nutritional Strategy of an Endurance Master Athlete Following Hip Arthroplasty: A Case Study

**DOI:** 10.3389/fspor.2020.00009

**Published:** 2020-02-13

**Authors:** Julien Louis, Eve Tiollier, Antonia Lamb, Bastien Bontemps, Jose Areta, Thierry Bernard

**Affiliations:** ^1^Research Institute for Sport and Exercise Sciences, Liverpool John Moores University, Liverpool, United Kingdom; ^2^French National Institute of Sport, Expertise and Performance, Sport, Expertise and Performance Lab, Paris, France; ^3^Research Unit “Impact of Physical Activity on Health (IAPS N°201723207F) University of Toulon, Toulon, France; ^4^Laboratoire Motricité Humaine, Education, Sport, Santé (LAMHESS), Université Côte d'Azur, Nice, France

**Keywords:** body composition, triathlon, aging, energy availability, macronutrients, performance, osteoarthritis

## Abstract

Retraining and resuming competition following surgery is challenging for athletes due to the prolonged period of reduced physical activity and subsequent alteration of body composition and physical performance. This is even more challenging for master athletes who endure the additional effect of aging. Within this context, the purpose of this study was to evaluate the feasibility and benefits that evidence-based nutritional and training recommendations could have on the time course of reconditioning and retraining following hip arthroplasty in an endurance master triathlete. During 38 weeks (from 6 weeks prior to surgery through to the return to competition in week 32), the athlete was provided with detailed training and nutritional recommendations. Dietary intake (via the remote food photographic method), body composition (via DXA), peak oxygen uptake (VO_2peak_), peak power output (PPO), cycling efficiency (GE), and energy availability (EA) were assessed 6 weeks pre- and 8, 12, 18, 21, and 25-weeks post-surgery. Training load was quantified (via TRIMP score and energy expenditure) daily during the retraining. Total body mass increased by 8.2 kg (attributable to a 3.5–4.6 kg increase in fat mass and lean mass, respectively) between week −6 and 8 despite a reduction in carbohydrate (CHO) intake post-surgery (<3.0 g/kg body mass/day). This was accompanied with a decrease in VO_2peak_, PPO, and GE due to a drop in training load. From week 7, the athlete resumed training and was advised to increase gradually CHO intake according to the demands of training. Eventually the athlete was able to return to competition in week 32 with a higher PPO, improved VO_2peak_, and GE. Throughout retraining, EA was maintained around 30 kcal/kg Lean Body Mass/day, protein intake was high (~2 g/kg/day) while CHO intake was periodized. Such dietary conditions allowed the athlete to maintain and even increase lean mass, which represents a major challenge with aging. Data reported in this study show, for the first time, the conditions required to recover and return to endurance competition following hip surgery.

## Introduction

Master athletes are defined as athletes >40 years old who maintain a high level of physical fitness despite the physiological effects of aging, allowing them to compete in sporting competitions (Reaburn and Dascombe, 2008). Participation of master athletes is steadily increasing, especially in endurance events such as marathons (Jokl et al., 2004), and triathlons (Bernard et al., 2010), with male master triathletes accounting for 48% of male finishers in Ironman triathlons during 2007–2010 (Stiefel et al., 2014). Master athletes strive to maintain performances they achieved at younger ages, even though athletic performance inevitably declines with aging (Lepers et al., 2013; Lepers and Stapley, 2016; Louis et al., 2020).

Regular physical activity throughout life can mitigate the age-related decline in muscle mass, muscle function, and cardiorespiratory capacity (Mian et al., 2007; Louis et al., 2009; Bieuzen et al., 2010). However, chronic high-level exercise as practiced by endurance master athletes may have adverse health implications including osteoarthritis (OA). OA is defined as the breakdown of joint cartilage and subsequently underlying bone as a result of joint inflammation (Friery, 2008). Intense training programs, increased biomechanical load (Radin et al., 1991), and repetitive high impact activities such as long-distance running as well as family history, are among the main factors responsible for OA (Friery, 2008). Although the precise relationship between training load and the risk of OA is not well-defined, many observational studies have reported an increased risk of knee and hip OA in former elite athletes (Lefevre-Colau et al., 2016). Team sports such as basketball, football, and hockey, that require many direction changes, present the highest risk of developing knee and hip OA (Lefevre-Colau et al., 2016). In endurance sports, the risk is lower but athletes still have a 1.73 increased chance of being admitted into hospital with severe OA, generally affecting lower limb joints (Kujala et al., 1994) at an average age of 59.7 years (Kettunen et al., 2001). The burden of OA is mainly caused by chronic pain, impaired function, and reduced quality of life (Cai et al., 2019).

The standard course of treatment for severe OA of the hip is a total arthroplasty which replaces the entire joint (Meira and Zeni, 2014) or resurfacing arthroplasty which replaces the surfaces of the hip joint affected by OA (Oxblom et al., 2019). Resurfacing arthroplasty has become the preferred treatment for athletes because it preserves more bone. Whatever the technique used, the surgery is often preceded and followed by a period of reduced activity and even immobilization of lower limbs due to pain sensations about the joint. In general, the treated lower extremity is immobilized after a hip arthroscopy for 6–8 weeks (Stalzer et al., 2006; Wahoff and Ryan, 2011). Such situation inevitably leads to a decrease in muscle mass, a possible reduction in bone mass, an increase in fat tissue, accompanied with metabolic, and functional alterations such as a loss in muscle strength (Houston et al., 1979; Suetta et al., 2009). The time period of ceased training also elicits reversibility of training adaptions that take considerably longer to re-establish than the time period of detraining (Houston et al., 1979). Furthermore, with the additional effect of aging, this may potentially result in the requirement of a longer reconditioning time period than that of younger athletes (Hvid et al., 2010). During immobilization and reconditioning, dietary intake also plays an important role and can support physiological adaptation for a quicker rehabilitation. Specifically, adapting energy intake to the changes in energy expenditure while maintaining a high protein intake can limit the decline in muscle mass (Louis et al., 2019a,b). Little is known on the time-course of reconditioning and return to competition of young endurance athletes following surgery, and even less is known about deconditioning and reconditioning of master athletes.

With this in mind, we present the retraining and nutritional strategy that allowed an endurance master triathlete to return to competition 32 weeks following a hip arthroplasty. Training load, energy intake, body composition, and aerobic capacity were recorded at regular intervals during the period. Specifically we were interested in the changes in lean mass and bone mineral content that endured the combined effects of reduced physical activity and aging.

## Materials and Methods

### Experimental Approach to the Problem

**Figure 1:**
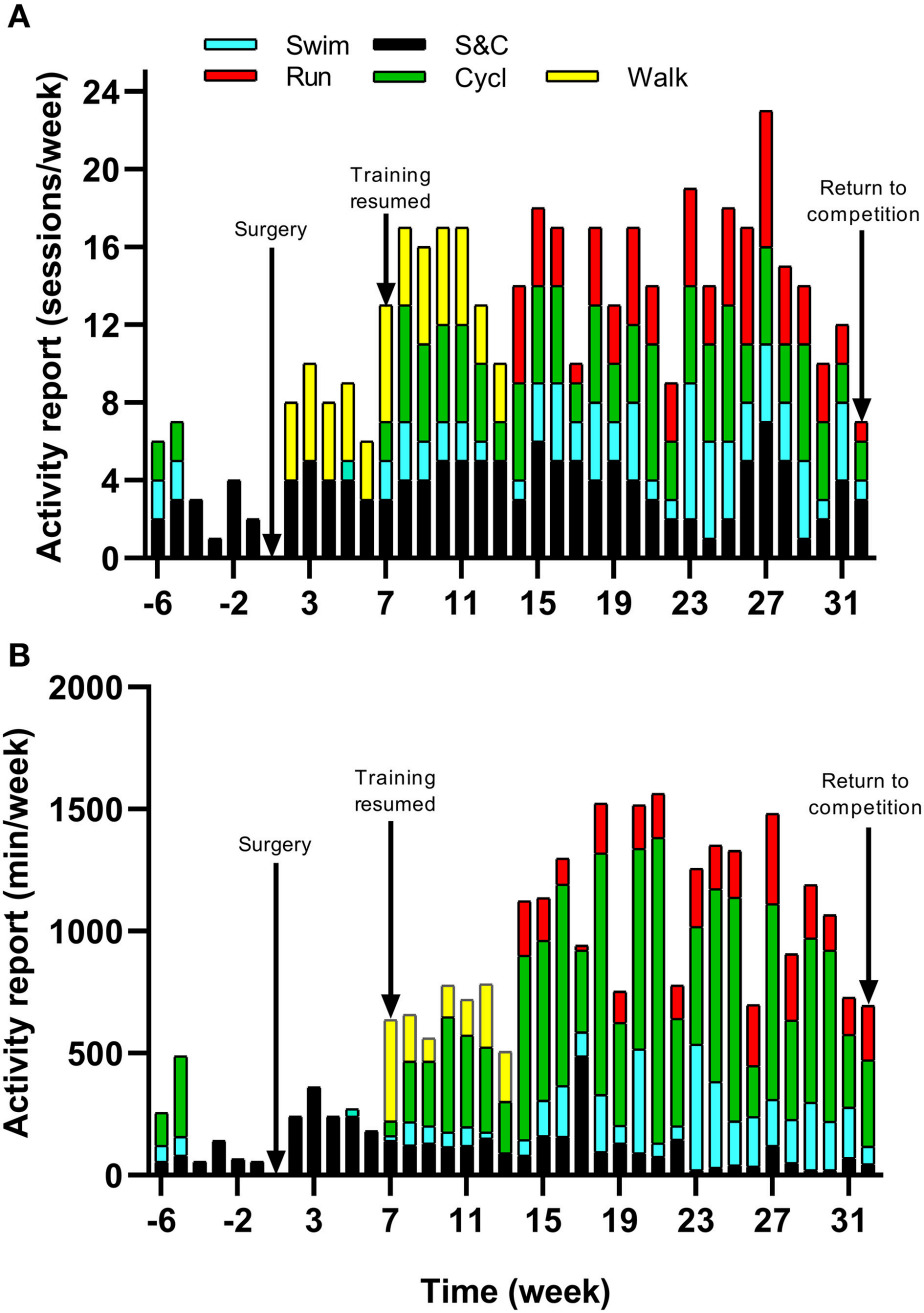
Timeline of physical activity and training undertaken with **(A)** Frequency and **(B)** Duration of strength and conditioning (S&C), walking (walk), swimming (swim), cycling (cycl), and running (run) sessions from week −6 to 32.

Because of increased pain after competing in the 70.3 Ironman World Championship in South Africa in September 2018 and diagnosis of osteoarthritis, the athlete underwent a resurfacing arthroplasty of the leg hip in November 2018. Surgery was performed 12 weeks after the last competition when pain was no longer bearable. In light of the previous research, this case study was therefore designed to observe the benefits that informed-based nutritional and training recommendations could have on the time course of reconditioning following hip arthroplasty. The pre-surgery assessment (i.e., body composition, cycling maximal aerobic capacity, daily energy intake, and expenditure) of the athlete was carried out 6 weeks following the last competition corresponding to 6 weeks prior to the surgery. Due to excessive pain, at the time of the first assessment (week −6), the athlete's daily activity was already dramatically reduced with only 2,737 ± 1,234 steps/day, 55 min of strength and conditioning, 65 min of swimming, and 135 min of cycling recorded during the week (Figure 1). In the last 4 weeks prior to the surgery, a worsening of pain even obliged the athlete to use a wheelchair on some days. The athlete was not completely immobilized at any point following the surgery but stayed 7 days post operation non-weight bearing on his injured leg with the help of crutches. Nutritional and physical training recommendations were provided to the athlete from the 1^st^ day following the surgery (week 1) until the end of the intervention (week 32). The athlete's physiotherapist also contributed to the design and implementation of the training program. Nutritional recommendations were adapted to the energetic demands of the athlete's physical activity on a weekly basis, while the training load was adapted to the sensations of the athlete. Physical testing sessions were organized regularly during the intervention (6 weeks prior to and 8, 12, 18, 21, and 25 weeks post-surgery) to evaluate the progress made by the athlete and to readjust the nutritional and physical training recommendations. The main investigator met the athlete on a monthly basis for testing sessions and spoke to the athlete on the phone on a weekly basis during the whole duration of the study to ensure compliance with the protocol and answer potential questions. The rationale for nutritional and training recommendations was systematically discussed.

### The Subject

The athlete is a 52-year-old master triathlete who competes in 5–6 triathlons (from Olympic to Ironman distance) per year. He regularly competes in the 70.3 Ironman age group World Championship. Over the last 3 years preceding the surgery, the athlete's yearly best performance on the 70.3 Ironman distance averaged +4.2 ± 0.9% of the winner time of his age group, equating to +11 ± 3 min. At the time of the surgery, the athlete's physical characteristics were: age 51 years old, body mass 83.4 kg and height 195 cm. The athlete had a training history of at least three training sessions per week in swimming, cycling, and running for the last 15 years. The study, methodology, and the possible health risks and benefits that could result from participation were explained to the athlete. The athlete voluntarily agreed to participate in this study and signed an informed consent for the publication of the data reported in this case study. The study was approved by the research ethics committee of Liverpool John Moores University. The protocol was in conformity with the declaration of Helsinki (last modified in 2013).

### Procedures

Six weeks prior to and 8, 12, 18, 21, and 25 weeks post-surgery, the athlete underwent the same series of tests. Caffeine and alcohol consumption were abstained in the 24 h preceding each testing session. After an overnight fast, the athlete visited the laboratory for a whole-body fan beam dual-energy X-ray absorptiometry (DXA) measurement scan (Hologic Discovery A, WA, USA) according to the methods described by Nana et al. (2012, 2013). Values of lean body mass (LBM), fat mass and bone mineral content (BMC) for the whole body and the treated leg were retained for analysis. Subsequently, the athlete performed a submaximal intensity cycling test, immediately followed by a step test until volitional exhaustion (Lepers et al., 2019). Oxygen uptake was continuously measured using indirect calorimetry via an automated open circuit system (Medgraphics, Ultima CariO_2_, MGC Diagnostics, Minnesota, USA). Heart rate (HR) was monitored via a Polar V800 heart rate monitor (Polar, Finland) and blood lactate (La) was recorded at the end of each cycling stage using a portable lactate analyzer (Lactate Pro2, Arkray, Japan). Gross efficiency (GE) and substrate (CHO and FAT) oxidation were determined from the submaximal intensity test (Jeukendrup and Wallis, 2005) while peak aerobic capacity (VO_2peak_) and peak power output (PPO) were obtained from the step test (Louis et al., 2012).

**Figure 2:**
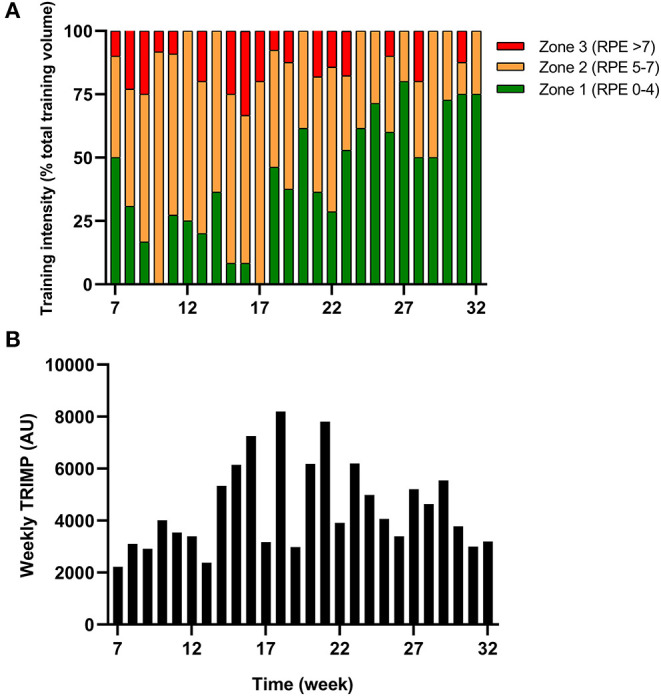
Training **(A)** intensity (according to RPE) and **(B)** load (expressed as TRIMP) during the period of retraining from week 7–32.

The athlete completed a detailed training diary (including duration, distance, session RPE, and HR) for strength & conditioning (S&C), swimming (Swim), cycling (Cycl), running (Run), and walking activities (Walk) at week −6, and then daily from post-surgery until competition in week 32. From week 1 (surgery week) to week 6, the athlete engaged in S&C (mainly isometric strength training), Walk and Swim sessions only, accompanied with physiotherapy sessions 3 times a week (including stretching, massage, and proprioception). Structured training (including S&C, Swim, and Cycl) resumed in week 7 and gradually increased until week 32 (Figure 1). Run sessions resumed in week 14 and gradually increased thereafter. During this period of structured training, training load was calculated as training impulse (TRIMP) by using session rate of perceived exertion (RPE, 1–10) and session duration (min) according to the method of Foster et al. (2001). Using RPE, three training intensity zones could be identified: Zone 1 = RPE 0–4 (v*ery easy to somewhat hard*); Zone 2 = RPE 5–7 (*hard*); and Zone 3 = RPE >7 (*very hard to maximal*) (Stellingwerf, 2012) (Figure 2). By monitoring HR and using the equation described by Crouter et al. (2008) we could quantify energy expenditure during training sessions (ExEE, kcal). Daily energy availability (EA, kcal/kg LBM) could therefore be estimated for the testing weeks during which dietary intake was also monitored (Heikura et al., 2018). In week −6, 1, 4, 8, 12, 18, 21, and 25, the athlete reported all food and fluid ingested for 7 consecutive days by using the remote photographic technique, so that energy intake (EI) and the distribution in macronutrients (CHO, FAT, and PRO) could be determined. In order to achieve energy balance during reduced ExEE (weeks 1–7), the athlete was advised to adopt a daily low CHO-high PRO diet (CHO <3.0 g/kg BM; PRO >2 g/kg BM; FAT; 1–1.5 g/kg BM). From week 7, the athlete was advised to increase his daily CHO intake (up to 8 g/kg BM in days with high intensity or long duration training sessions) according to the training load, while PRO, and FAT intake were maintained. In order to maximize the anabolic processes, the athlete was recommended to ingest 30–40 g of protein at each meal and snacks (i.e., every 3–4 h) (Louis et al., 2019b). Dietary protein sources were the preferred choice while protein based supplements were consumed as snacks on some occasions. The athlete was also recommended to ingest a casein protein based supplement before sleep (Groen et al., 2012). Daily nutritional plans were provided to the athlete (Table 1). A breakdown of actual energy and macronutrient intake is presented in Table 2.

**Table 1:** Examples of dietary meal plans provide to the athlete during the immobilization and rehabilitation/return to training period.

**Meal (Time)**	**Immobilization phase**	**Rehabilitation/training phase**
Breakfast (7 a.m.)	3 fried eggs + 1 avocado + 1 slice brown bread + 1 fresh orange	1 medium banana + 200 ml orange juice + porridge (with 100 g oat flakes, 28 g honey, 250 ml semi-skimmed milk, 40 g mixed nuts and raisins)
Morning snack (10 a.m.)	200 g yogurt + 15 blueberries	40 g Whey protein powder with 250 ml water + 1 pear
Lunch (1 p.m)	200 g mixed salad with olive oil + 1 medium chicken breast without skin (120 g) + 80 g boiled courgettes + 160 g boiled basmati rice + 250 ml semi-skimmed milk	200 g boiled pasta + 1 tablespoon olive oil + 1 medium chicken breast without skin (120 g) + 80 g boiled courgettes + 140 g fruit salad
Afternoon snack (4 p.m.)	40 g Whey protein powder + 250 ml semi-skimmed milk	1 medium banana + 150 ml apple juice + 40 g Whey protein with 250 ml water
Dinner (7 p.m.)	200 g mixed salad with olive oil + 1 tomato + 1 average salmon darn + 160 g protein rich yogurt	200 g mixed salad with olive oil + 200 g boiled basmati rice + 1 average salmon darn + 115 g baguette bread + 150 ml apple juice + 1 Greek style fruit yogurt (125 g)
Evening snack (10 p.m., approx. 30–60 min before sleep)	40 g Casein protein powder + 250 ml semi-skimmed milk	40 g Casein protein powder + 250 ml semi-skimmed milk
Approximate daily macronutrient intake	2,305 kcal: 151 g CHO, 207 g PRO, and 97 g FAT	3,352 kcal: 440 g CHO, 200 g PRO, and 88 g FAT
Approximate daily macronutrient intake (relative to body weight)	27.1 kcal/kg: 1.8 g/kg CHO, 2.4 g/kg PRO, and 1.2 g/kg FAT	39.4 kcal/kg: 5.2 g/kg CHO, 2.3 g/kg PRO, and 1 g/kg FAT

*Nutritional information calculated with a nutrition analysis software (Nutritics, Research Edition, Dublin, Ireland)*.

**Table 2:** Daily exercise energy expenditure, energy intake, energy availability, carbohydrate, lipid, and protein intake during each testing week.

	**Week 6**	**Week 1**	**Week 4**	**Week 8**	**Week 12**	**Week 18**	**Week 21**	**Week 25**
ExEE (kcal/kg LBM)	7.9 ± 7.7	3.7 ± 2.0	12.2 ± 3.4	13.5 ± 6.0	32.3 ± 12.9	28.5 ± 9.2	28.0 ± 9.1	30.6 ± 15.0
EI (kcal/kg LBM)	54.4 ± 13.6	40.3 ± 6.3	44.3 ± 6.8	45.6 ± 8.1	57.2 ± 15.0	55.1 ± 5.4	54.6 ± 6.7	60.5 ± 16.8
EA (kcal/kg LBM)	46.5 ± 17.8	36.6 ± 4.9	32.1 ± 9.1	32.1 ± 8.2	24.9 ± 21.6	26.6 ± 10.7	26.6 ± 12.6	29.9 ± 21.3
CHO (g/kg BM)	4.5 ± 0.8	2.7 ± 0.3	3.0 ± 0.2	3.5 ± 0.7	4.8 ± 1.2	5.0 ± 1.0	5.0 ± 0.8	6.1 ± 2.2
CHO (as % of EI)	43.1 ± 7.0	35.3 ± 3.8	36.9 ± 7.3	39.0 ± 4.0	43.8 ± 7.4	44.3 ± 6.6	45.2 ± 3.8	48.8 ± 8.2
FAT (g/kg BM)	2.0 ± 0.8	1.3 ± 0.3	1.7 ± 0.5	1.5 ± 0.3	1.7 ± 0.8	1.6 ± 0.3	1.6 ± 0.3	1.8 ± 0.6
FAT (as % of EI)	39.6 ± 9.3	38.7 ± 5.1	44.1 ± 12.7	37.7 ± 5.7	33.3 ± 7.3	31.4 ± 5.1	33.1 ± 4.9	33.4 ± 8.4
PRO (g/kg BM)	1.7 ± 0.5	2.0 ± 0.4	1.8 ± 0.3	2.2 ± 0.7	2.4 ± 0.6	2.6 ± 0.3	2.4 ± 0.3	2.2 ± 0.6
PRO (as % of EI)	15.7 ± 2.9	26.0 ± 3.4	21.8 ± 2.3	23.4 ± 4.1	21.9 ± 3.2	23.7 ± 3.2	21.3 ± 2.0	17.8 ± 1.3

*LBM, Lean Body Mass; BM, Total Body Mass; CHO, Carbohydrates; FAT, Lipids; PRO, Proteins*.

## Results

**Figure 3:**
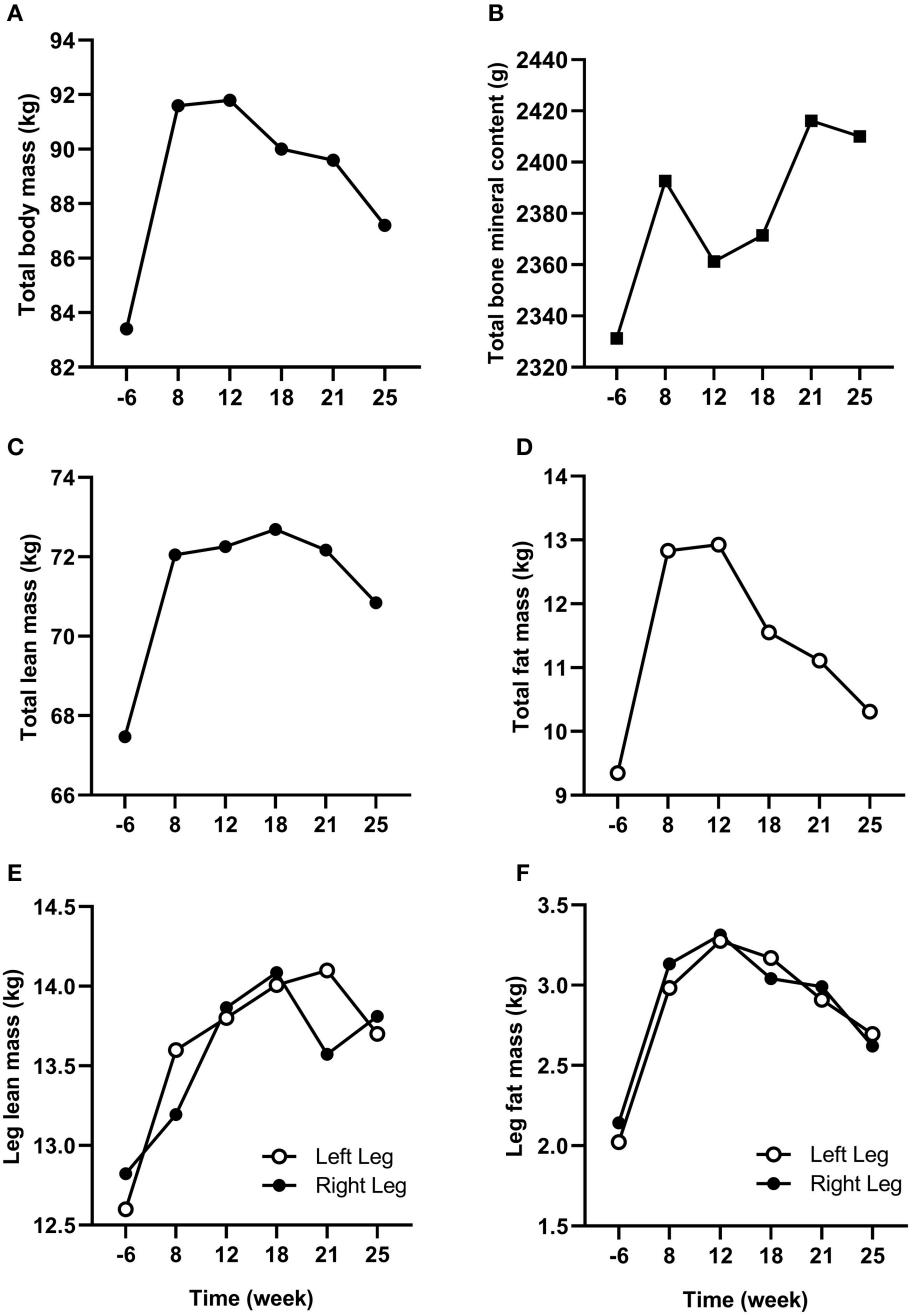
Changes in total **(A)** body mass, **(B)** bone mineral content, **(C)** lean mass, and **(D)** fat mass from week −6 to 25. Changes in **(E)** lean mass and **(F)** fat mass for the injured (left) and non-injured (right) leg from week −6 to 25.

Changes in body composition are presented in Figure 3. Between week −6 and 8 which corresponded to the period of low ExEE, total body mass increased by 8.2 kg. It was attributable to a 3.5 and 4.6 kg increase in fat mass and lean mass, respectively. Lean mass and fat mass of the injured leg presented a similar evolution to total body over the period. Interestingly no decrease in lean mass was recorded in week 8 despite low EA (close to 30 kcal/kg LBM/day) post operation (Table 2). In the successive 17 weeks, body mass gradually decreased along with fat mass, while lean mass remained higher than pre-surgery. A high PRO intake associated with periodized CHO and EI according to the demands of training likely contributed to this positive outcome. In addition, thanks to a gradual increase in training load between weeks 7 and 32 (Figure 2), EA was gradually reduced likely contributing to the decrease in fat mass (Table 2). Total bone mineral content (BMC) was not negatively altered during the period and even increased (+3.4%) from week −6 to 25 (Figure 3).

The cycling test results of VO_2peak_, PPO, HR, [La], substrate oxidation, and GE are presented in Table 3. Compared to week −6, VO_2peak_ (in ml/min/kg) was reduced by 31.4, 14.3, 9.5, 7.7, and 13.5% in week 8, 12, 18, 21, and 25, respectively. However, the decrease in VO_2peak_ was lower when excluding body mass from the calculation (in L/min) with a decrease of 21.2, 6.4, 2.1, 6.4, and 10.6% in week 8, 12, 18, 21, and 25, respectively, showing that aerobic capacity was almost recovered. PPO was recovered from week 18 (405 W) and even improved by 8.6% (440 W) and 17.3% (475 W) in weeks 21 and 25.

**Table 3:** Physiological data recorded during the cycling tests during each testing week.

	**Week 6**			**Week 8**			**Week 12**			**Week 18**			**Week 21**			**Week 25**		
**Intensity (W)**	**135**	**240**	**405**	**135**	**240**	**275**	**135**	**240**	**345**	**135**	**240**	**405**	**135**	**240**	**440**	**135**	**240**	**475**
VO_2_ (L/min)	1.7	3.1	4.7	2.1	3.4	3.7	2.1	3.3	4.4	1.9	3.0	4.6	1.8	2.5	4.4	1.8	3.0	4.2
VO_2_ (ml/min/kg)	20.8	37.3	56.1	23.4	37.4	38.5	22.9	36.5	48.1	20.9	32.9	50.8	21.1	32.9	51.8	20.8	34.3	48.5
VO_2_ (ml/min/kg LBM)	25.8	46.1	69.4	29.7	47.6	51.1	29.2	46.3	61.1	25.8	40.7	62.9	24.7	34.8	60.7	25.6	42.2	59.7
VO_2_ (% VO_2peak_)	37	66	100	58	93	100	48	76	100	41	65	100	41	57	100	43	71	100
HR (beats/min)	102	136	163.0	100	131	136	99	133	154	99	127	154	93	119	156	88	117	156
[La] (mmol/L)	1.3	1,7	4.7	1.2	2.2	2.5	1.3	1.9	3.2	2.3	2.4	4.7	1.9	1.3	4.3	2.3	1.5	4.2
CHO oxidation (g/min)	1.31	2.35		1.69	3.85		1.11	2.01		1.09	1.45		1.39	2.33		1.47	2.10	
FAT oxidation (g/min)	0.31	0.62		0.38	0.15		0.60	0.88		0.49	0.89		0.33	0.44		0.31	0.64	
Gross efficiency (%)	22.7	22.5		18.4	20.0		19.0	23.8		21.2	26.2		22.0	26.2		21.6	23.5	

*VO_2_, oxygen uptake; LBM, lean body mass; HR, heart rate; [La], blood lactate concentration; CHO, carbohydrates; FAT, lipids. CHO and FAT oxidation rates were determined by using the equation developed by Jeukendrup and Wallis (2005). VO_2_ values for submaximal intensities (135–240 W) correspond to the mean of the last minute of each 5 min stage. VO_2_ values for maximal sustained intensity corresponding to peak power output (PPO) correspond to the 30 s interval containing the two highest 15 s O_2_ consumption value. The highest intensity (W) corresponds to PPO which is the highest power output sustained for 1 min*.

## Discussion

Overall, the rehabilitation and retraining intervention was a success as the athlete was able to return to competition 32 weeks after surgery. At the end of the intervention, lean mass and BMC were improved, though fat mass was still higher than pre-surgery. The athlete recovered and even improved his cycling PPO, though VO_2peak_ was still lower than pre-surgery. Running was possible as the athlete completed an average of 189 ± 73 min per week between weeks 14 and 32, though the athlete required more time to increase the training volume and be entirely competitive in the discipline.

The main challenge faced by the athlete was to maintain his body composition that endured the cumulated effects of low physical activity for several weeks and the catabolic effects of aging (Rolland et al., 2008). Bed rest studies, albeit an extreme form of immobilization, have reported an average decrease of ~0.5% of total muscle mass per day of immobilization and the effect was even accentuated for lower limbs compared to upper limbs (Wall et al., 2013, 2014). In our study, as expected, fat mass increased between week −6 and 8 due to high EI compared to ExEE (EA = 46.5 ± 17.8 kcal/kg LBM/day in week −6) pre surgery. During this time, FAT intake was also high (corresponding to 39.6, 38.7, and 44.1% of total EI in week −6, 1, and 4, respectively) which could contribute to the increase in fat mass. However, lean mass concomitantly increased thanks to the combination of increased daily PRO intake and regular S&C training as soon as in week 2 (Mettler et al., 2010). Such conditions, likely provided an increased anabolic stimulus for muscle protein synthesis despite the comparatively low EA (25–27 kcal/kg LBM/day) between weeks 1 and 25 (Areta et al., 2014). Furthermore, PRO consumption every 3–4 h was recommended during the entire period to maximize the stimulation of muscle protein synthesis throughout the day (Areta et al., 2013). However, dietary analyses revealed that only 59, 85, and 69% of morning, afternoon, and bedtime PRO snacks, respectively, were consumed, suggesting that daily PRO intake >2.0 g/kg BM may be of greater importance than consuming PRO every 3–4 h in maintaining lean mass in master athletes. Another positive outcome of this study was that BMC increased from pre to post-surgery (Walters et al., 2012). Conversely to our results, Mueller et al. (2015) reported a decline in BMC of the injured leg in the first 2 months following hip arthroplasty due to femoroacetabular impingement in a 32-year old professional female Ironman triathlete. BMC returned to pre-surgery values after 6 weeks of rehabilitation. The quicker return to pre-surgery BMC values in our study may be explained by an earlier return to full weight bearing activities because mechanical loading of bone is the main driver of bone formation (Buehlmeier et al., 2017). Indeed, in our study the athlete only stayed 7 days post operation non-weight bearing on his injured leg with the help of crutches whereas it lasted 4 weeks in Mueller et al. (2015) study. The periodization of nutritional intake to maintain energy availability around 30 kcal/kg LBM/day and a high protein intake throughout the different phases of the rehabilitation also likely contributed to the maintenance of BMC in our athlete (Sale and Elliott-Sale, 2019).

The regular monitoring of cycling aerobic capacities showed that VO_2peak_ (in ml/min/kg) dropped by 31.4% in week 8 which corresponded to only 21.2% when expressed in absolute value (in L/min). Hence, it appears that the increase in body mass and more specifically non-contractile tissue (fat mass) greatly impacted maximal aerobic capacity. This rapid decrease in VO_2peak_ following training cessation is also generally explained by cardiac mechanisms (i.e., decrease in stroke volume), while peripheral mechanisms (i.e., decrease in mitochondrial enzyme activity) appear more slowly in athletes with numerous years of endurance training (Coyle et al., 1984; Nichols et al., 1997). The overall pattern of retraining from week 8 to 25 showed a steady improvement in VO_2peak_ and PPO concomitant with an improvement in GE. Surprisingly in week 25 the athlete reached 117% of pre-surgery PPO likely explained by an increased muscle mass with >2 kg located in the legs. Substrate oxidation also gradually returned to baseline with the contribution of fat and carbohydrates to total energy production increasing and decreasing, respectively, from weeks 8 to 25. Unfortunately, the absence of data from other master athletes prevent the comparison with other studies.

Return to sport after hip arthroplasty is becoming common expectation for patients, though return to competition-level sport can be challenging. In a 4.7 years' prospective study, Girard et al. (2017) recently reported that 94% of patients returned to sport by 4 months post-arthroplasty, but only 58% of them returned to competition-level sport (i.e., long distance triathlon in this study). This was mainly explained by the fear of early wear despite 100% implant survival rate. In the present study, the athlete returned to competition in week 32 (IronMan 70.3, in Europe). He placed 1^st^ in his age group in swimming and 3^rd^ in cycling, but could not maintain the pace in the running part of the triathlon (123^th^ in running and 82^th^/155 overall, +25% of race winner time). In week 45, the athlete took part in another competition (IronMan 70.3, in Europe) and placed 1^st^ in swimming, 4^th^ in cycling, and 45^th^ in running, finishing in 9^th^ place/155 overall equating +4% of race winner time. This confirms the retraining data showing that master triathletes can return to competition 7–10 months after hip resurfacing arthroplasty but more time is required to retrieve their full potential in running.

Overall, this case study provides a positive outcome that must be considered in light of its limitations. As in all case study, the first limitation is that only one master athlete was studied, hence additional studies are necessary to verify whether this pattern of retraining would be consistent in other master athletes. Due to logistical constraints, no body composition and physiological measurements were possible closer to the cessation of training, surgery time and return to competition. Nevertheless, we hope the data will provide guidance to practitioners and athletes who must deal with periods of forced reduced physical activity due to surgery or injury.

## Conclusion

This case study was conducted as a real-world applied example for master athletes and practitioners seeking to optimize rehabilitation/retraining programs through to the return to competition following hip arthroplasty. More specifically these data inform about the timeline of deconditioning and reconditioning following surgery and how it can be optimized through adapted nutrition and physical training. Our main findings suggest that endurance master athletes can counteract the combined deleterious effects of prolonged periods of muscle disuse and aging on body composition and physical capacity. This requires the avoidance of total immobilization, an early return to physical activity in the form of S&C sessions and the maintenance of daily energy availability around 30 kcal/kg LBM.

## Data Availability Statement

All datasets generated for this study are included in the article/supplementary material.

## Ethics Statement

The study was approved by the research ethics committee of Liverpool John Moores University. The protocol was in conformity with the declaration of Helsinki (last modified in 2013). The patients/participants provided their written informed consent to participate in this study.

## Author Contributions

The study was designed by JL and TB. Data were collected by JL, TB, AL, and BB. Data were analyzed by JL, AL, TB, JA, and ET. Data interpretation and manuscript preparation were undertaken by JL, AL, ET, BB, JA, and TB. All authors approved the final version of the article.

### Conflict of Interest

The authors declare that the research was conducted in the absence of any commercial or financial relationships that could be construed as a potential conflict of interest.
